# Retrospective analysis of comprehensive nursing care management in pyoderma gangrenosum: Case series and literature review

**DOI:** 10.1097/MD.0000000000045415

**Published:** 2025-10-24

**Authors:** Qiannan Cao, Yaqiong Ma, Shiguang Peng

**Affiliations:** a Department of Dermatology, Beijing Chao-yang Hospital, Capital Medical University, Beijing, China.

**Keywords:** comprehensive nursing, pain control, psychological care, Pyoderma gangrenosum, wound management

## Abstract

**Rationale:**

Pyoderma gangrenosum (PG), a rare immune-mediated dermatosis characterized by painful, necrotic, and rapidly progressive ulcers, is often associated with systemic diseases such as inflammatory bowel disease and rheumatoid arthritis, posing significant clinical management challenges. This study aimed to evaluate the efficacy of comprehensive nursing strategies for PG patients through retrospective analysis, summarize clinical nursing experiences, and provide references for improving patient outcomes.

**Patient concerns:**

Clinical data from 7 PG patients admitted to Beijing Chaoyang Hospital, Capital Medical University, between January 2014 and December 2024 were retrospectively analyzed.

**Diagnoses:**

Seven cases were diagnosed with PG according to the Delphi expert consensus criteria.

**Interventions:**

All patients received systemic immunosuppressive therapy (primarily glucocorticoids, with some combined with immunosuppressants) and multidimensional nursing interventions (including ward management, wound care, pain control, comorbidity monitoring, psychological care, and health education).

**Outcomes:**

Following systemic treatment and comprehensive nursing, all 7 patients achieved significant ulcer healing. The average pain score decreased from 7.86 ± 1.07 at admission to 0.86 ± 0.90 at discharge, with no severe complications reported. Individualized wound management (e.g., topical triethanolamine cream) and strict infection control were critical to therapeutic success. However, 2 cases developed secondary infections due to suboptimal ward isolation.

**Lessons:**

Within the limitations of this small retrospective case series, our findings suggest that multidimensional comprehensive nursing interventions may serve as a beneficial adjunct to systemic medical therapy for PG patients, potentially contributing to wound healing, pain alleviation, and complication reduction. However, the observed outcomes cannot be definitively attributed solely to nursing care due to the lack of a control group. Future prospective, multicenter, and controlled studies with larger cohorts are essential to validate the efficacy and cost-effectiveness of these comprehensive nursing strategies. Nonetheless, optimizing protective isolation measures remains a critical practical consideration to mitigate infection risk in these vulnerable patients.

## 1. Introduction

Pyoderma gangrenosum (PG) is a rare, chronic, recurrent skin ulcerative disease with a peak incidence between 40 and 60 years of age and an incidence of only a few cases per million person-years.^[1]^ It can affect individuals of any age. The initial rash is usually an inflammatory papule, vesicle, pustule, or nodule, which progresses to a central necrotic ulcer that deepens and expands, accompanied by severe pain. The most characteristic clinical feature is an irregular, undermined, necrotic, and painful ulcer, which heals with a cribriform, pigmented, atrophic scar.^[2]^ PG ulcers are often deep and exposed, prone to secondary infection. The lesions are extremely painful, and approximately 75% of patients have associated systemic diseases, such as inflammatory bowel disease, inflammatory arthritis, and hematological disorders.^[3]^ This leads to complex clinical manifestations. Studies have shown that the mortality rate for PG patients is 3 times higher than that of the general population matched for age and sex.^[4]^ Proper care is essential for PG patients. Inappropriate care can increase the risk of secondary infection, delay or worsen wound healing, prolong pain, exacerbate complications, and lengthen hospital stays. Therefore, comprehensive nursing care is crucial for preventing secondary infections, promoting wound healing, alleviating patient suffering, and shortening the disease course.^[5]^ A retrospective analysis has been conducted on the clinical data of 7 patients with PG admitted to our hospital between January 2014 and December 2024. The findings of this analysis are reported below.

## 2. Materials and methods

### 2.1. Study population

Inclusion criteria: cases diagnosed with PG according to the Delphi expert consensus criteria;^[6]^ hospitalized at Beijing Chaoyang Hospital, Capital Medical University, between January 2014 and December 2024. Exclusion criteria: non-initial admissions to our hospital; primary hospitalization diagnoses unrelated to PG or undocumented PG-specific nursing interventions. A retrospective review was conducted on cases meeting the inclusion criteria while excluding those falling under the exclusion criteria. Clinical manifestations, treatment strategies, nursing protocols, and disease outcomes were systematically analyzed. All procedures conducted in this study adhere to the ethical standards outlined in the 1964 Helsinki Declaration and its subsequent amendments or comparable ethical guidelines, and have been approved by the Beijing Chaoyang Hospital IRB (No. 2023-R-351). The members of this research team contacted the patient or their registered representative by phone to obtain authorization to use their clinical data for this retrospective analysis and publication, followed by obtaining written informed consent.

### 2.2. Statistical analysis

Data were analyzed using SPSS 28.0 (IBM Corp, Armonk, NY). Continuous variables are presented as median and mean ± standard deviation. For statistical analysis of patients’ pain score, Self-Rating Anxiety Scale (SAS) and Self-Rating Depression Scale (SDS) score, paired samples *t*-tests were employed to evaluate differences between admission and discharge measurements, as these constituted paired observations. The null hypothesis stated that no significant difference existed in mean scores between admission and discharge. A two-tailed significance level of *α* = 0.05 was applied. To facilitate interpretation, effect sizes were estimated using Cohen *d* along with their corresponding 95% confidence intervals for paired comparisons.

## 3. Results

### 3.1. Patient demographics

**Figure 1.**
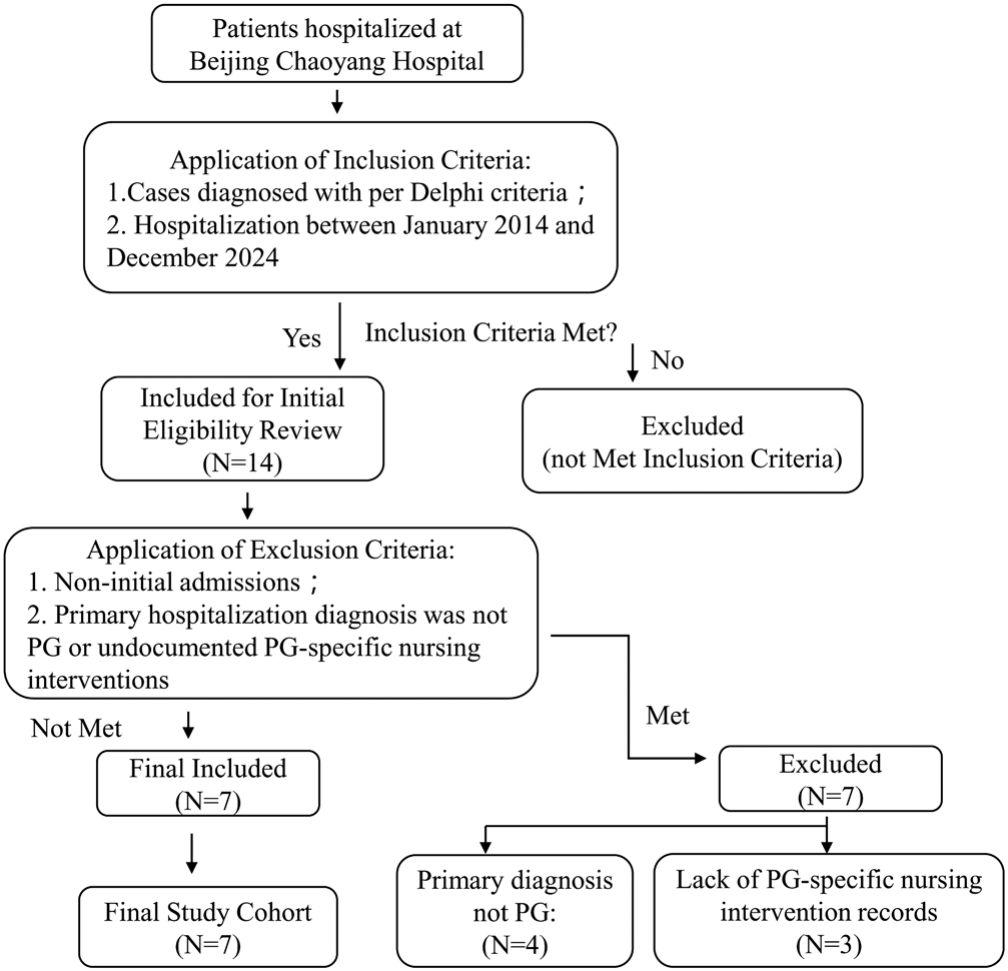
Patient screening flowchart.

Between January 2014 and December 2024, 14 hospitalized patients initially met the inclusion criteria.^[7]^ Of these, 7 were excluded based on predefined exclusion criteria (Fig. 1). The final cohort comprised 7 patients (3 males, 4 females) with a mean age at onset of 50.0 ± 14.5 years (range: 23–66; median: 52 years). The mean disease duration at admission was 30.7 ± 33.0 months (range: 2–84; median: 9 months), and the average hospital stay was 23.0 ± 7.7 days (range: 13–37; median: 22 days).

### 3.2. Clinical manifestations

**Figure 2.**
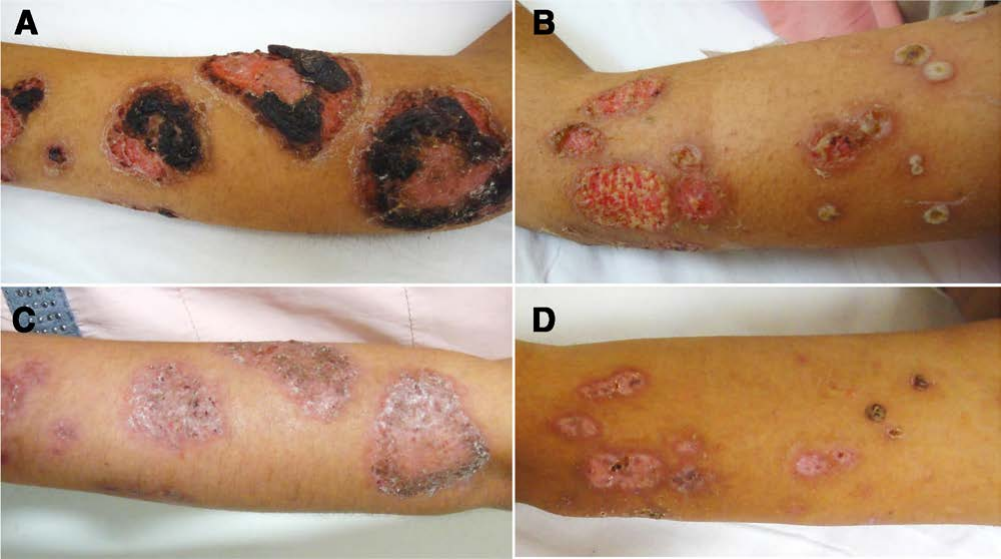
Representative patient’s skin lesions at admission and discharge after treatment. (A) Skin lesion on the left forearm at admission; (B) skin lesion on the left upper arm at admission; (C) left forearm skin lesion at discharge; (D) left upper arm skin lesion at discharge.

All patients presented with spontaneous-onset lesions. Six cases exhibited ulcerative lesions, while one had pustulo-ulcerative lesions. Lower extremity involvement was universal (n = 7), with specific distributions as follows: isolated lower limbs (n = 2), combined upper and lower limbs (n = 1), trunk and lower limbs (n = 2), trunk and all limbs (n = 1), and concurrent facial, trunk, and limb involvement (n = 1) (Fig. 2). All lesions were associated with severe pain. Bacterial cultures taken from the ulcer sites of 2 patients tested positive: 1 patient, who also had diabetes, tested positive for *Enterobacter cloacae* and *Acinetobacter baumannii*, while the other patient, who suffered from ulcerative colitis and chronic hepatitis B, tested positive for *Klebsiella pneumoniae*. Comorbidities included ulcerative colitis (n = 1), severe anemia (n = 1), type 2 diabetes (n = 1), hypertension (n = 2), hyperlipidemia (n = 1), and cardiac insufficiency (n = 1) (Table 1).

**Table 1:** Clinical characteristics of 7 patients with pyoderma gangrenosum.

No.	Gender	Age	Disease course (mo)	Location of lesions	Type of lesions	Bacterial culture of lesions	Comorbidities	Systemic treatment strategy	Hospitalization days	Outcome
1	Male	61	8	Lower limbs	Ulcerative	Negative	Hypertension, cerebral infarction	Hydrocortisone 200mg qd, minocycline 100mg bid	22	Improved and discharged
2	Male	43	9	Abdomen, lower limbs	Ulcerative	Klebsiella pneumoniae	Ulcerative colitis, hepatitis B	Methylprednisolone 20mg qd	23	Improved and discharged
3	Male	46	4	Lower limbs	Ulcerative	Negative	Hyperlipidemia, colon polyps	Prednisone 20mg bid	18	Improved and discharged
4	Female	52	60	Lower limbs, chest	Ulcerative	Negative	Nutritional chronic anemia, old tuberculous meningitis	Methylprednisolone 40mg qd, mycophenolate mofetil 0.5 bid	28	Improved and discharged
5	Female	66	48	Back, chest, abdomen, limbs, vulva	Ulcerative	Enterobacter cloacae, Acinetobacter baumannii	Diabetes	Methylprednisolone 40mg qd + prednisone 20mg qd, minocycline 100mg bid	37	Improved and discharged
6	Female	23	84	Limbs, trunk, face	Ulcerative, pustular	Negative	Cardiac insufficiency	Methylprednisolone 30mg qd, minocycline 100mg bid	13	Improved and discharged
7	Female	59	2	Limbs	Ulcerative	Negative	Hypertension	Prednisone 20mg qd	20	Improved and discharged

bid = twice daily, mo = month, qd = once daily.

### 3.3. Treatment strategy

Prior to admission, several patients had been misdiagnosed with cutaneous infections, panniculitis, or eczema at external institutions and received ineffective therapies, including oral antihistamines and topical glucocorticoids. Following definitive diagnosis at our hospital, all 7 patients were initiated on systemic glucocorticoids. The daily prednisone-equivalent dosage ranged from 20 to 75 mg (median: 40 mg). Adjunctive therapies included minocycline 100mg twice daily (n = 3) and mycophenolate mofetil 0.5 g twice daily (n = 1). All patients underwent standardized wound care protocols, including regular dressing changes, management of secondary infections, pain severity assessments (using the numerical rating scale, NRS), and tailored analgesic therapy.

### 3.4. Multidimensional nursing interventions

All 7 patients presented with extensive skin lesions, severe pain, and comorbid systemic diseases, resulting in complex clinical management. In addition to systemic therapy, a comprehensive nursing strategy was implemented to enhance recovery and alleviate symptoms. Specific interventions included:

(1) Ward management: Patients were assigned to 3-bed rooms, positioned in the innermost window-side beds, separated by privacy screens to minimize cross-exposure. No infectious disease patients were cohoused. Daily air disinfection was performed using a mobile UV unit (equipped with two 30W UV lamps, intensity > 70 μW/cm² at 1.0 m) for 30 minutes. Strict measures were implemented to shield patients from UV exposure during disinfection. Rooms were ventilated for 20 to 30 minutes twice daily (morning and evening) while ensuring patient warmth. Visitor access was strictly limited in duration and number to maintain cleanliness and reduce infection risks.
(2) Basic care: Patients with ulcerative colitis or severe anemia meeting critically ill criteria were assigned Level I nursing care, characterized by continuous physiological monitoring with 30-minute assessments and hourly vital signs documentation. This level prioritizes life-sustaining interventions and surveillance for acute deterioration. The remaining 5 patients received Level II nursing care, involving 2-hourly evaluations and quad-daily vital signs recording, with emphasis on functional recovery support and complication prevention during convalescence. Autoclaved linens, bedding, and hospital garments were provided individually and replaced daily. Ethylene oxide-sterilized disposable under-pads were placed beneath patients and changed promptly based on wound exudate volume.
(3) Wound care: Wound care served as a critical adjunct to systemic PG therapy.^[8,9]^ Given the heterogeneous nature of PG lesions, individualized strategies were implemented based on ulcer type:
  A. Pustular PG:

- Debridement: Deep debridement was avoided to prevent trauma-induced exacerbation.
- Topical management: Daily 10-minute compresses with 0.5% potassium permanganate solution were applied to lesions. Anti-inflammatory halometasone cream and prophylactic mupirocin ointment were used to mitigate bacterial colonization.
- Post-pustular care: After complete desiccation and crust formation, petroleum jelly ointment was applied to facilitate gentle crust removal.

B. Ulcerative PG:
  - Infection surveillance: Regular bacterial/fungal microscopy and culture of ulcer secretions guided targeted anti-infective therapy.
  - Necrosis management: Superficial necrotic tissue was gently debrided. Pus at the ulcer base was removed using ethacridine lactate-soaked cotton swabs, preserving underlying granulation tissue.
  - Antimicrobial dressing: A gentamicin–saline solution (80,000 IU:250 mL) was applied via sterile gauze moist compresses for 20 minutes per session.
  - Granulation promotion: After compresses, triethanolamine cream was thickly applied to the ulcer base to stimulate granulation, followed by sterile gauze coverage.
  - Monitoring: Daily documentation included ulcer size, secretion characteristics, exudation levels, and epithelial migration. Dressing frequency was reduced to every other day once purulent discharge ceased and exudate minimized to non-penetrative levels, continuing until full healing.

(4) Pain management: Pain intensity was assessed using the NRS.^[10]^ For patients with NRS scores ≤ 5, nonpharmacological interventions were prioritized, including breathing exercises, physical analgesia (e.g., cold/heat therapy), and distraction techniques. For those with NRS scores > 5, a combination of nonpharmacological and pharmacological approaches was employed. Pharmacotherapy included nonsteroidal anti-inflammatory drugs (NSAIDs) or opioids, with pain reassessment every 2 to 3 hours post-administration until NRS scores fell below 5. Patient satisfaction with pain management was evaluated daily, and adjustments to analgesic regimens were made in collaboration with clinicians as needed.
(5) Comorbidity and complication monitoring: PG patients often present with systemic comorbidities (e.g., ulcerative colitis, type 2 diabetes, hypertension) and face elevated risks of complications such as hyperglycemia, hepatorenal dysfunction, osteoporosis, and secondary infections due to prolonged glucocorticoid/immunosuppressant use. In this cohort, comorbidities included ulcerative colitis (n = 1), severe anemia (n = 1), type 2 diabetes (n = 1), hypertension (n = 2), cardiac insufficiency (n = 1), and hyperlipidemia (n = 1). Nursing interventions focused on:
  - Monitoring: Regular assessment of urine/stool characteristics, frequency, and volume; tracking blood glucose, complete blood counts, and liver/kidney function.
  - Preventive measures: Fall prevention protocols and environmental safety checks.
  - Collaborative care: Coordinating with physicians to manage comorbidities through targeted therapies (e.g., anti-hypertensives, insulin adjustments).
(6) Psychological care: Psychological care is integral to comprehensive nursing. Effective psychological support helps patients develop a realistic understanding of their condition, fosters confidence in recovery, enhances compliance with medical care, and ultimately reduces disease duration and suffering. Due to disease recurrence, severe pain, and high treatment costs, PG patients often experience varying degrees of anxiety and depression.^[11]^ Anxiety and depression levels were assessed using the SAS and SDS. Nurses provided individualized psychological interventions through regular communication to alleviate distress. For severe anxiety/depression, pharmacotherapy was administered under physician guidance.
(7) Health education: Patient education and communication are critical for PG management.^[12]^ Upon admission, all patients received structured education covering disease progression, treatment plans, and prognosis. Dietary recommendations emphasized high protein, high vitamin, low-fat, and low-salt intake, with carbohydrate adjustments based on glycemic control. Patients were instructed to promptly report symptom recurrence during or after hospitalization and to attend scheduled follow-ups.

### 3.5. Disease outcomes

For patients with comorbid ulcerative colitis, critical care status was downgraded to Level II nursing once skin ulcer area reduced by 50%, granulation tissue became prominent, and purulent discharge resolved. Similarly, patients with severe anemia transitioned to Level II care after anemia correction, ulcer cleansing, and epithelial coverage exceeding 50% of the ulcer area. Following specialized treatment and comprehensive nursing interventions, all 7 patients achieved progressive skin lesion healing, significant pain relief, and improved mental status. Statistically significant reductions were observed in clinical scores: Mean pain NRS scores decreased from 7.86 ± 1.07 at admission to 0.86 ± 0.90 at discharge (*P* < .0001), while mean SAS scores decreased from 72.71 ± 11.20 to 53.14 ± 9.96 (*P* < .0001), and mean SDS scores decreased from 59.29 ± 10.94 to 50.29 ± 8.93 (*P* = .0012) (Table 2).

**Table 2:** Pain NRS score, SAS and SDS scores of 7 patients with pyoderma gangrenosum.

No.	Gender	Age	Pain NRS score		SAS score		SDS score	
			On admission	At discharge	On admission	At discharge	On admission	At discharge
1	Male	61	7	0	58	42	52	41
2	Male	43	7	0	66	44	50	42
3	Male	46	7	0	62	48	46	42
4	Female	52	8	1	80	64	64	54
5	Female	66	10	1	88	68	70	65
6	Female	23	8	2	83	56	75	58
7	Female	59	8	2	72	50	58	50
Mean ± SD			7.86 ± 1.07	0.86 ± 0.90	72.71 ± 11.20	53.14 ± 9.96	59.29 ± 10.94	50.29 ± 8.93
*P*-value			<.0001		<.0001		.0012	
Cohen *d*			2.54		1.87		0.91	
95% CI for *d*			[1.24–3.84]		[0.76–2.98]		[0.18–1.64]	

CI = confidence interval, NRS = numerical rating scale, SAS = self-rating anxiety scale, SDS = self-rating depression scale.

## 4. Discussion

PG is a rare immune-mediated dermatosis characterized by rapidly progressive, necrotic ulcers with undermined borders. PG patients often exhibit immunosuppression due to both the disease itself and systemic immunomodulatory therapies, predisposing them to diverse complications and complex clinical presentations. Additionally, psychological comorbidities are prevalent, further challenging comprehensive management.

Systemic therapy remains the cornerstone of PG treatment, particularly for severe, extensive, or refractory cases. First-line options include corticosteroids and immunosuppressants such as cyclosporine A, azathioprine, and mycophenolate mofetil. Recent advances in understanding PG pathophysiology have expanded therapeutic avenues, with biologics (e.g., anti-TNF agents, IL-1 receptor antagonists, anti-IL-1β, anti-IL-6R, anti-IL-12/23, anti-IL-17) and small-molecule inhibitors (e.g., tofacitinib, baricitinib, ruxolitinib) increasingly utilized in clinical practice.^[13]^

Local therapy is a vital component of PG management, serving either as monotherapy for small, localized lesions or as an adjunct to systemic treatments to enhance efficacy and mitigate adverse effects. Mainstay topical agents include corticosteroids and calcineurin inhibitors.^[14]^ Additionally, sporadic case reports describe the use of gels or solutions containing cyclosporine, dapsone, methotrexate, cromolyn sodium, phenytoin, infliximab, or etanercept for PG treatment.^[15]^ In our cohort, triethanolamine cream was applied to ulcer sites alongside systemic therapy to maintain a moist wound environment conducive to granulation tissue growth, aligning with modern moist wound healing principles. Triethanolamine, a safe stabilizer and pH regulator widely used in skincare products, is likely to promote angiogenesis, wound contraction, and accelerate healing.^[16]^ These beneficial effects are likely attributable to its stabilizing and pH-modulating properties.

Comprehensive nursing care plays a pivotal role in the recovery of patients with PG. For the 7 cases in this study, individualized care plans were developed alongside nurse-led wound management, tailored to address each patient’s unique clinical profile. Key components included:

- Ward management: Optimizing environmental hygiene and infection control protocols.
- Pain management: Utilizing validated scales (e.g., NRS) to guide analgesic strategies.
- Comorbidity monitoring: Coordinating with physicians for conditions such as diabetes and hypertension.
- Psychological support: Implementing anxiety/depression screening (SAS/SDS) and counseling.
- Patient education: Providing dietary guidance and follow-up instructions.

These interventions probably collectively enhanced therapeutic outcomes and alleviated patient suffering. However, limitations in ward infrastructure – specifically, the inability to provide single-bed rooms – led to secondary bacterial infections in 2 cases. Both patients had complications that increased their vulnerability to secondary infections: 1 with ulcerative colitis and chronic hepatitis B, and the other with diabetes. To mitigate infection risk, protective isolation in single-bed rooms should be implemented wherever possible, particularly for high-risk patients with comorbid conditions that heighten infection susceptibility. When limited ward resources preclude single-room isolation, patients should be placed in window beds farthest from the ward entrance, with strict enforcement of protective isolation measures.

## 5. Limitations

This study has several limitations that should be acknowledged. First, the small sample size (n = 7) limits the statistical power and generalizability of the findings, which is a common challenge in studies of rare diseases such as PG. Second, retrospective design introduces potential biases, including missing data, recall bias, and inaccuracies in subjective measurements – particularly those related to pain and psychological assessments. Third, the absence of a control group hinders our ability to attribute outcomes solely to nursing interventions; future studies should consider using historical controls or propensity score matching where feasible.

Additionally, practical constraints such as the use of shared wards – which contributed to an increased risk of infection in 2 cases – highlight confounding factors that may affect the evaluation of nursing outcomes. The resource intensity of comprehensive nursing was not quantitatively analyzed in relation to clinical outcomes; a cost-effectiveness analysis would be valuable in future research. Furthermore, long-term recurrence rates and the sustained effects of nursing interventions beyond hospitalization were not assessed, which are important for evaluating the durability of care.

Prospective, multicenter studies with larger sample sizes and controlled designs are needed to validate the efficacy of comprehensive nursing strategies in the management of PG.

## Acknowledgments

We sincerely thank all the healthcare professionals in the dermatology ward for providing exceptional care to PG patients, and we are also grateful to all the patients who have kindly agreed to let us use their clinical information.

## Author contributions

**Conceptualization:** Shiguang Peng.

**Data curation:** Qiannan Cao, Shiguang Peng.

**Investigation:** Qiannan Cao, Yaqiong Ma, Shiguang Peng.

**Methodology:** Qiannan Cao, Yaqiong Ma, Shiguang Peng.

**Resources:** Qiannan Cao.

**Supervision:** Yaqiong Ma, Shiguang Peng.

**Validation:** Shiguang Peng.

**Writing – original draft:** Qiannan Cao, Shiguang Peng.

**Writing – review & editing:** Qiannan Cao, Yaqiong Ma, Shiguang Peng.
